# Drug interaction alerts in older primary care patients, and related medically justified actions

**DOI:** 10.1007/s00228-022-03292-4

**Published:** 2022-03-30

**Authors:** Carina Tukukino, Naldy Parodi López, Staffan A. Svensson, Susanna M. Wallerstedt

**Affiliations:** 1 Department of Pharmacology, Sahlgrenska Academy, University of Gothenburg, Box 430, 405 30 Gothenburg, Sweden; 2 Department of Clinical Pharmacology, Sahlgrenska University Hospital, Gothenburg, Sweden; 3 Närhälsan Kungshöjd Health Centre, Gothenburg, Sweden; 4 Närhälsan Hjällbo Health Centre, Gothenburg, Sweden; 5 HTA-Centrum, Sahlgrenska University Hospital, Gothenburg, Sweden

**Keywords:** Drug-drug interaction alert, Interaction database, Medication therapy management, Older people, Polypharmacy, Primary care

## Abstract

**Purpose:**

To describe presented interaction alerts in older patients, and the extent to which these require further medical action for the specific patient or are already being addressed.

**Methods:**

Interaction alerts presented at a physician consultation, for 274 consecutive primary care patients treated with two or more drugs (median age: 75 years; 59% female), were extracted. These alerts are based on *J*anusmed, a decision support integrated in the medical records that provides recommendations for managing the interactions. One general practitioner (GP) and one GP/clinical pharmacologist determined in retrospect, first independently and then in consensus, whether the alerts justified further medical action, considering each patient’s health condition.

**Results:**

In all, 405 drug interaction alerts in 151 (55%) patients were triggered. Medical action in response was deemed medically justified for 35 (9%) alerts in 26 (17%) patients. These actions most often involved a switch to a less interacting drug from the same drug class (*n* = 10), a separate intake (*n* = 9), or the ordering of a laboratory test (*n* = 8). Out of 531 actions suggested by the alert system, only 38 (7%) were applicable to the specific patient, as, for instance, laboratory parameters were already being satisfactorily monitored or a separate intake implemented.

**Conclusions:**

More than every other older patient receives drug treatment that triggers drug interaction alerts. Nine in ten alerts were already being addressed or were not relevant in the clinical setting, whereas, for the remaining tenth, some medical action, that for unknown reasons had not been taken, was reasonable. These findings show that interaction alerts are questionable as indicators of problematic prescribing.

## Introduction

As patients are being treated with an increasing number of drugs [1], the risk of drug interactions is rising and interactions have been described as a significant cause of hospital visits and admissions [2]. It is, however, difficult to estimate the significance of drug interactions in clinical practice, as studies often report prevalence figures based solely on hits in interaction databases, and only rarely attempt to assess actual harm and other medical outcomes of interactions [3, 4].

Several web-based electronic databases have been developed to identify potentially problematic drug interactions [5, 6], forming the basis for clinical decision support systems [7–9]. Such systems have been shown to be useful during the patient consultation [10]. On the other hand, decision support systems pose a risk of alert fatigue, previously described as the mental state resulting from too many alerts consuming time and mental energy, which can cause important alerts to be ignored along with clinically unimportant ones [11]. A systematic review reported that up to 71% of hospitalised patients have potential drug interactions [3], and another review reported that the prevalence of clinically manifested interactions in hospitalised patients ranged from 1 to 64% [4]. Given these figures, it may not be surprising that up to 95% of drug interaction alerts have been reported to be overridden by clinicians [12]. There are various reasons for overriding an alert, one being that the potential interaction problem has already been taken care of. Unless an automatic decision support system is made aware of this by integration with patient data, it will keep on presenting alerts as long as the interacting drugs remain in the medication list. The extent to which automatically generated interaction alerts are already managed in health care, and details of such management, is an under-researched topic.

Given that the prevalence of drug interactions is used scientifically to reflect drug treatment quality, as illustrated by the recent inclusion of this item in core outcome sets for improving prescribing [13, 14], and, further, that the purpose of interaction alerts is to aid clinical decision making, there is a need for increased knowledge on interaction alerts in clinical practice. In this study, we aimed to shed light on interaction alerts in older patients, and the extent to which these require further action or are already being adequately addressed by the patient’s physician.

## Methods

This descriptive study was conducted using data from a previous study investigating the association between medication reviews (recorded by a procedure code) and the adequacy of drug treatment management, in 302 consecutive patients (≥ 65 years of age) with a planned physician consultation at one of two Swedish primary care centres in the autumn of 2017 [15]. In that study, the drug treatment of each patient was retrospectively assessed by two specialist physicians (N.P.L., general practitioner (GP); S.S., GP/clinical pharmacologist), based on printouts from the electronic medical records over the 2½ years preceding the consultation, including laboratory tests, hospital discharge records, vaccinations, prescriptions, as well as interaction alerts originating from the Swedish national interaction database *J*anusmed [7]. The assessors determined whether an action related to the drug treatment was medically justified, prior to the next regular visit (see Appendix 1 for sentences guiding their categorisation). The assessments were performed from an overall medical perspective, first independently and then jointly where disagreements were resolved through consensus.

In the current study, patients with fewer than two drugs in the medication list were excluded; drugs for topical use were only counted if having potential systemic effects. Drugs used regularly or pro re nata (PRN) were considered. We recorded presented *J*anusmed category *B*, *C* and *D* interaction alerts: *B*, an interaction where the clinical relevance is uncertain or varies; *C*, a clinically relevant interaction that can be managed by either dose adjustments or separated intake; and *D*, a clinically relevant interaction where the recommendation is to avoid the drug combination [16]. These alerts are automatically presented in the prescribing module of the electronic health record system, as buttons highlighted in white, yellow and red, respectively. Therefore, the prescribing physicians were exposed to, and had the chance to react to, the same alerts as did the assessors. We also entered each patient’s current medication list into the open-access interface of *J*anusmed (January 2020) [17] to retrieve the specific recommendations provided to manage the alerts [7].

Additional medically justified actions prior to the next regular visit were recorded if they were related to the interaction alerts, as determined retrospectively by the assessors in consensus. For instance, this could include the switch or withdrawal of a drug, the ordering of a laboratory test, the retrieval of more information about the patient, or arranging an extra visit. Dosing was considered in the assessments.

Patients’ characteristics included age, sex, residence, and morbidities appearing in the Screening Tool of Older Persons’ Prescriptions (STOPP), the Screening Tool to Alert to Right Treatment (START), or the Swedish set of indicators of prescribing quality provided by the National Board of Health and Welfare [18, 19]. Multi-dose drug dispensing, i.e. machine-dispensed unit bags with drugs that are ingested at specific times of the day, intended for patients with difficulties in handling their drugs, has been associated with *D* interactions [20]. Therefore, we also recorded whether the patient was using this system or not.

## Ethics approval

The study was approved by the Regional Ethical Review Board in Gothenburg, Sweden (DRN: 1046–15).

## Statistics

We performed descriptive analyses using SPSS for Windows, version 24.0 (IBM SPSS, Armonk, NY, USA). The inter-rater agreement was assessed using kappa statistics.

## Results

**Fig. 1:**
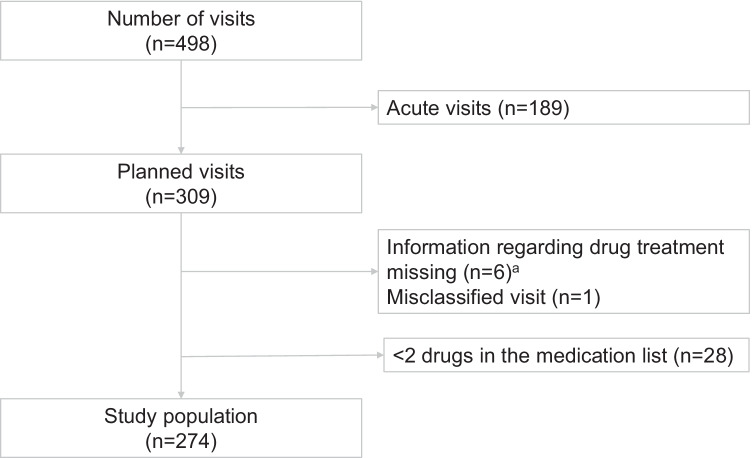
Flowchart of the study population, starting from all recorded visits by individuals ≥ 65 years of age to either of two primary health care centres, 9 Oct–5 Nov 2017. ^a^Deceased patients with multi-dose drug dispensing where information regarding drug treatment could not be retrieved after death

In all, 274 patients were included in the analysis (Fig. 1, Table 1). The median age was 75 years and 163 (59%) were women. The patients were treated with a median of seven drugs, ranging from two to 20. A total of 33 out of 274 (12%) patients were using multi-dose drug dispensing.

**Table 1:** Characteristics of patients (*n* = 274). Values are presented as n (%) or median (range)

Age, yrs			75 (65–99)
Female			163 (59)
Multi-dose drug dispensing			33 (12)
Residing in a nursing home			30 (11)
Medication list	Regular	Number of drugs	5 (1–17)
		≥ 5 drugs	153 (56)
	PRN	Number of drugs	2 (1–8)
		≥ 1 drug	196 (72)
Common morbidities	Hypertension		197 (72)
	Osteoarthritis		84 (31)
	Type 2 diabetes		84 (31)
	Insomnia		73 (27)
	Chronic ischaemic heart disease		56 (20)
	Depression		54 (20)
	Impaired cognition, including dementia		38 (14)
	eGFR < 60 mL/min, last year		65 (24)

*eGFR* estimated glomerular filtration rate, *PRN* pro re nata

The interaction decision support system presented 405 drug interaction alerts, encompassing 185 unique drug-drug combinations, in 151 (55%) patients (Table 2). Up to 13 drug combination alerts were detected in a single individual. Overall, an additional action was deemed medically justified for 35 (9%) alerts in 26 patients (9% of all patients, 17% of patients with one or more interaction alerts). For 349 (86%) of 405 alerts, the assessors made the same assessment regarding whether a related action was medically justified or not, resulting in a kappa value of 0.44.

**Table 2:** Number and type of interaction alerts in 274 older primary care patients with two or more drugs in their medication list, according to the *J*anusmed interaction database, as well as the extent to which it, in retrospect, was deemed medically justified to act on these alerts for a specific patient

	**Interaction alert level**^a^		**Patient level**	
*Janusmed category*^*b*^	*Total* *n*	*Related action medically justified *^*c*^ *n (% of all alerts in the corresponding category)*	≥ *1 interaction alert* *n (% of all patients)*	≥ *1 related action medically justified *^*c*^ *n (% of patients with* ≥ *1 interaction alert in the corresponding category)*
B	199	0	113 (41)	0
C	197	31 (16)	101 (37)	22 (22)
D	9	4 (44)	8 (3)	4 (50)

^a^Patients with interactions in two or more different categories are included in each group

^b^*B* clinical interaction where the clinical relevance is uncertain or varies, *C* clinically relevant interaction that can be managed by dose adjustments or separated intake, *D* clinically relevant interaction where the recommendation is to avoid the drug combination

^c^An interaction alert for which it was deemed, in retrospect, to be medically justified, considering the condition of the specific patient, to perform additional medical action prior to the next regular physician visit

Joined to the 405 alerts, *J*anusmed provided a total of 531 recommendations, 38 (7%) of which were judged applicable to the specific patient (Table 3). None of the recommendations to monitor clinical signs, to perform therapeutic drug monitoring (TDM), and to add a proton pump inhibitor (PPI) required any action. Other specific *J*anusmed recommendations were applicable in 2–40% of the cases in which they were provided.

**Table 3:** Recommendations for clinical management (*n* = 531) provided in 405 *J*anusmed interaction alerts, triggered in 274 older primary care patients with two or more drugs in their medication list, and the number of alerts where a corresponding action, in retrospect, was considered medically justified prior to the next regular physician visit

*Recommendation*	*Total* *n (% of alerts)*^*a*^	*Related action medically justified* *n (% of alerts with the corresponding recommendation)*
Adjust dose	54 (13)	1 (2)
Avoid the drug combination	25 (6)	4 (16)^f^
Monitor clinical signs^b^	18 (4)	0
Monitor clinical parameters^c^	48 (12)	1 (2)
Perform TDM	31 (8)	0
Monitor laboratory parameters^d^	165 (41)	8 (5)
Switch to another drug	106 (26)	11 (10)^f^
Add a PPI	24 (6)	0
Separate the intake	50 (12)	9 (18)
Vigilance^e^	10 (2)	4 (40)
No action required	23 (6)	N/A

*N/A* not applicable, *PPI* proton pump inhibitor, *TDM* therapeutic drug monitoring

^a^Includes any recommendation provided in *J*anusmed for the drug combination alert

^b^Changed effects or adverse effect

^c^Blood pressure, electrocardiogram (ECG), heart rate, weight

^d^Sodium and potassium levels, renal or liver function tests

^e^Caution suggested regarding the drug combination, check indication, contact the patient’s physician, e.g. their cardiologist

^f^Including one case where the indication for treatment first had to be considered

Medically justified actions related to drug interactions primarily concerned switching to a less interacting drug with the same mechanism of action (*n* = 10), separating the intake (*n* = 9), ordering a laboratory test (*n* = 8), or searching for more information before decision making (*n* = 5) (Table 4). Interaction alerts where a related action was medically justified frequently involved omeprazole (*n* = 9), (es)citalopram (*n* = 9), clopidogrel (*n* = 6), ferrous sulphate (*n* = 5), spironolactone (*n* = 4) or furosemide (*n* = 3). Four of these included a drug used PRN (diclofenac, *n* = 3; codeine, *n* = 1).

**Table 4:** Description of interaction alerts which were presented in three or more patients and/or which, in retrospect, warranted further medical action

Interaction pair	*J*anusmed alert^a^		Physician assessment^b^	
	*n*	*Described medical consequence*	*Action*	*No action, reason*
*D interactions*				
Clopidogrel–repaglinide	1	Increased exposure to repaglinide, with increased risk of hypoglycaemia	Check medication list with patient’s cardiologist (*n* = 1)	(*n* = 0)
Codeine–paroxetine	1	A marked decrease of the analgesic and antitussive effect of codeine	Switch to another SSRI (*n* = 1)	(*n* = 0)
Cholestyramine–warfarin	1	Impaired absorption, and therefore decreased effect, of warfarin	Check if indication persists, consider stopping cholestyramine (*n* = 1)	(*n* = 0)
Sotalol–verapamil	1	Increased risk of atrioventricular block, bradycardia, and severe hypotension	Retrieve medical charts from the cardiologist, re-evaluate treatment (*n* = 1)	(*n* = 0)
ASA–warfarin	1	Increased risk of bleeding; both substances interfere with the blood coagulation through different mechanisms	(*n* = 0)	Plaque indication according to the cardiologist (*n* = 1)
Citalopram–hydroxyzine	1	Additive prolonged effect on QT time	(*n* = 0)	Hydroxyzine PRN (*n* = 1)
Digoxin–verapamil	1	Increased exposure to digoxin with increased risk of toxicity	(*n* = 0)	Digoxin levels monitored regularly (*n* = 1)
Phenobarbital–fentanyl	1	Decreased fentanyl concentration; both substances increase the risk of respiratory depression	(*n* = 0)	Palliative care (*n* = 1)
Phenobarbital–oxycodone	1	Decreased oxycodone concentration; both substances increase the risk of respiratory depression	(*n* = 0)	Palliative care (*n* = 1)
*C interactions*				
Alendronate– calcium	12	Decreased absorption of bisphosphonate, with risk of insufficient effect	(*n* = 0)	Patient informed to separate the intake (*n* = 9) Verify separated intake at next visit (*n* = 3)
Calcium–levothyroxine	12	Decreased absorption of levothyroxine may reduce the effect slightly	Recommend separated intake (*n* = 3)	Stable TSH (*n* = 6) Verify separated intake at next visit (*n* = 3)
Furosemide–SSRI	12	The combination may cause hyponatraemia	Monitor sodium levels (*n* = 3)	Furosemide PRN (*n* = 3) Stable sodium levels (*n* = 4) Recently in hospital care (*n* = 1) Verify indication for furosemide and SSRI at next visit (*n* = 1)
Levothyroxine–PPI	11	Long-term treatment with a PPI decreases absorption of levothyroxine	(*n* = 0)	TSH regularly monitored (*n* = 11)
Paracetamol^c^–warfarin	9	Continuous use of paracetamol doses exceeding 2 g/day may increase the risk of bleeding	(*n* = 0)	INR regularly monitored (*n* = 9)
ASA–SSRI/SNRI	9	Combined with SSRI, low-dose ASA increases the risk of GI bleeding 5–7 times, and high-dose ASA 11–15 times	(*n* = 0)	Already on gastroprotection with a PPI (*n* = 5) No sign of bleeding (*n* = 3) Consider stopping ASA or starting a PPI at next visit (*n* = 1)
Omeprazole–(es)citalopram	8	Plasma concentration of (es)citalopram may increase (50–100%), with an increased risk of QT prolongation and therefore Torsade de pointes	Switch to pantoprazole (*n* = 5)	Verify indication for a PPI at next visit (*n* = 1) Verify indication for citalopram at next visit (*n* = 1) Low-dose citalopram (*n* = 1)
Clopidogrel–SSRI	6	Increased risk of bleeding	(*n* = 0)	Treated with a PPI (*n* = 3) Regular check-ups (*n* = 2) Monitor patient at next visit (*n* = 1)
ARB–diclofenac	5	Decreased antihypertensive effect and increased risk of renal failure	(*n* = 0)	Diclofenac PRN (*n* = 5)
ARB–spironolactone	5	Additive reduction of renal elimination of potassium, with increased risk of cardiac arrhythmia	Increase the monitoring of potassium (*n* = 3) Hyperkalaemia, check if the cardiologist monitors potassium (*n* = 1)	Normal potassium level and ECG (*n* = 1)
Calcium–ferrous sulphate	4	Decreased absorption of ferrous sulphate	Inform patient about separated intake (*n* = 2)	Monitor dosage regimen at next visit (*n* = 2)
Clopidogrel–omeprazole	4	The effect of clopidogrel may decrease	Switch to pantoprazole (*n* = 4)	(*n* = 0)
Levothyroxine–warfarin	4	Thyroid hormones may increase warfarin sensitivity	(*n* = 0)	INR is regularly monitored (*n* = 3) INR is regularly monitored at the coagulation clinic (*n* = 1)
Prednisolone–warfarin	4	Increased risk of GI bleeding, particularly in individuals with previous GI bleeding; increased INR value has been reported	(*n* = 0)	Already on gastroprotection with PPI, and INR regularly monitored at the coagulation clinic (*n* = 4)
Antacids–ferrous sulphate	3	Decreased absorption of ferrous sulphate	Separate the intake (*n* = 1)	Verify separated intake at next visit (*n* = 2)
Diclofenac–metoprolol	3	NSAID can in some patients decrease the antihypertensive effect of beta-adrenergic receptor antagonists	(*n* = 0)	Diclofenac PRN (*n* = 2) Episodic use (*n* = 1)
Simvastatin–warfarin	3	Increased effect of warfarin may occur; increased risk of bleeding	(*n* = 0)	Stable warfarin dose, INR regularly monitored (*n* = 3)
Amiloride–diclofenac	2	NSAID may impair the diuretic and antihypertensive effect; acute kidney failure may occur; the combination increases the risk of stomach ulcers	Stop diclofenac (*n* = 1)	Diclofenac PRN (*n* = 1)
Diclofenac–SSRI	2	Markedly increased risk of GI bleeding	Stop diclofenac (*n* = 1)	Already on gastroprotection with a PPI (*n* = 1)
Ferrous sulphate–levothyroxine	2	Decreased effect of levothyroxine	Separate the intake (*n* = 2)	(*n* = 0)
Antacids–levothyroxine	1	Decreased absorption of levothyroxine	Separate the intake (*n* = 1)	(*n* = 0)
Chlorzoxazone–simvastatin	1	Rhabdomyolysis and cholestasis may occur	Stop chlorzoxazone (*n* = 1)	(*n* = 0)
Clopidogrel–pioglitazone	1	Increased exposure to pioglitazone, with increased risk of hypoglycaemia and other dose-related adverse effects	Halve the dose of pioglitazone (*n* = 1)	(*n* = 0)
Diclofenac–enalapril	1	Impaired antihypertensive effect, increased risk of renal impairment	Check blood pressure and eGFR (*n* = 1)	(*n* = 0)
Gemfibrozil–magnesium	1	Gemfibrozil concentration may decrease by 50%	Check indication for magnesium, and consider a switch to a statin (*n* = 1)	(*n* = 0)

*ARB* angiotensin II receptor blocker, *ASA* acetylsalicylic acid, *ECG* electrocardiogram, *eGFR* estimated glomerular filtration rate, *GI* gastrointestinal, *INR* international normalised ratio, *NSAID* non-steroidal anti-inflammatory drug, *PPI* proton pump inhibitor, *PRN* pro re nata; *SNRI* serotonin–norepinephrine reuptake inhibitor, *SSRI* selective serotonin reuptake inhibitor, *TSH* thyroid-stimulating hormone

^a^*J*anusmed: *C* = clinically relevant interaction that can be managed by dose adjustments or separated intake; *D* = clinically relevant interaction where the recommendation is to avoid the drug combination

^b^Consensus decision by two specialist physicians, in retrospect, concerning whether further related medical action prior to the next regular physician visit was considered justified or not

^c^Acetaminophen in the USA

A total of nine *D* interaction alerts were presented in eight (3%) patients, all with unique drug combinations. For four of these alerts, encountered in four (1%) patients, some additional action was deemed medically justified (Table 4). Two actions involved consulting the patient’s cardiologist, one concerned a suggestion to switch to a less interacting drug within the same pharmacological subgroup, and one involved the withdrawal of a drug.

Category *C* interaction alerts were presented in 101 (37%) individuals. For 31 (16%) out of a total of 197 alerts, in 22 (8%) individuals, some additional action was considered medically justified. A frequent reason for the assessment that no action was needed, was that at least one of the drugs was used PRN (41 alerts in 26 individuals). Another frequent reason was ongoing monitoring of relevant laboratory parameters with adequate findings, for instance thyroid-stimulating hormone (TSH) in interactions involving levothyroxine (20 out of 23 alerts); international normalised ratio (INR) in interactions involving warfarin (20 out of 20 alerts); or electrolytes in drug combinations affecting sodium and/or potassium levels (ten out of 17 alerts). In addition, patients had usually already been informed about separated intake (25 out of 32 alerts). None of category *B* interaction alerts were considered to warrant medical action.

In 26 (79%) out of 33 patients using multi-dose drug dispensing, with a mean of twelve drugs in the medication list (range: four to 20), a total of 44 *C*, and 34 *B* interaction alerts were presented. In all, the assessors considered additional action to be medically justified in ten (13%) of these 78 alerts, all in the *C* category and encountered in seven (21%) patients. These actions involved a change to pantoprazole (*n* = 5), separated intake (*n* = 3), and the ordering of a laboratory test (*n* = 2).

## Discussion

In this study, we show that more than every second older patient with two or more drugs in their medication list has drug treatment that causes alerts in a well-established drug interaction decision support system. Only one alert out of eleven was considered medically justified to act upon prior to the next regular visit; in these cases, the prescribing physician had, for unknown reason, not taken action. The remaining alerts were either already being addressed or were not relevant in the clinical setting.

One interpretation of our results is that the interaction alerts integrated as a decision support tool have had the intended effect, that is, to affect clinicians' behaviour. For instance, the monitoring of laboratory parameters and TDM seem to be well managed in most cases, as seems to be the monitoring of clinical parameters including blood pressure, electrocardiogram and weight. Similarly, recommendations to adjust the dose, a strategy to mitigate potentially adverse consequences caused by drug interactions, merited action in merely one out of 50 alerts providing such advice. Our findings are in tune with previous research demonstrating that the prevalence of *D* interaction alerts decreased following the introduction of the interaction alert system used in the present study [16]; that interaction alerts are appropriately overridden in up to 84% of cases [12]; and that fewer than one in ten hospitalised patients are exposed to a clinically manifested drug interaction [4].

Only a minority of the alerts were deemed to merit additional action. Recommendations for management were often already attended to or not relevant in the specific case. As the alerts and recommendations continue to appear although the drug treatment is adequately managed, physicians may disregard them, thereby increasing the risk that important alerts are overlooked in a time-strained practice. To avoid information overload, one may hypothesize that the interaction alert system could benefit from increased integration with clinical data. For instance, although information sources may not be unambiguous [21], it could be valuable to incorporate dosing in the decision support system. In our study, for example, three out of nine omeprazole/(es)citalopram alerts did not require any action as a low dose was used. Further, two out of five *D* interaction alerts did not necessitate any action as at least one of the drugs was used PRN. On the other hand, our results illustrate that drugs used PRN may indeed be involved in drug interaction alerts where a related action is medically justified. Therefore, it may be problematic to reduce the alerts by simply applying a filter that makes the decision support system include only drugs prescribed regularly and in certain doses. However, one possibility could be to allow prescribers to temporarily disable interaction alerts already considered for a specific patient.

Alerts of the most serious category (*D*), where the recommendation is to avoid the drug combination, were shown in about one out of 30 patients. This prevalence of *D* interaction alerts is similar to the 3.8% reported in a previous study [22]. However, whereas the latter study was population-based, our study was restricted to patients 65 years or older with at least two drugs in their medication list. In addition, that study analysed drugs dispensed over a 4-month period, while we analysed the drugs actually included in a medication list after a planned consultation with a GP. Our study shows that less than half of the *D* alerts were medically warranted to act upon for the specific patient. Therefore, the prevalence of problematic *D* interactions in the general population may be considerably lower than indicated by prior research. The complexity of decision making is illustrated by our finding that one single action suggested in response to a *D* interaction concerned a simple switch from one drug to another within the same pharmacological subgroup. By contrast, all other actions due to *D* interactions required more complex clinical considerations.

Warfarin has been reported to be the drug most frequently seen in *D* interaction alerts [22]. Given the potentially severe consequences of interactions involving this substance, it is reassuring that, in our results, only one out of 26 *C/D* alerts including warfarin required medical action. In this case, the main issue was to check if an indication for the interacting agent (cholestyramine) persisted. In no case was the regular monitoring of INR problematic; such monitoring is well established in Sweden, with patients spending a high fraction of time in the therapeutic range [23]. Our results support findings in a previous study; integrating patient data, for instance laboratory parameters, may reduce alert severity in a non-negligible proportion of the interaction alerts, thereby increasing the alert specificity and decreasing the alert burden [24].

Almost half of the alerts were related to *C* interactions. Frequent *C* alerts concerned one drug affecting the absorption of another. Most of these alerts did not require any further medical action as the patient had already been informed to separate the intake. Almost all cases where the suggested action was a switch to another drug concerned substituting pantoprazole for omeprazole, which has been associated with a higher risk of drug interactions [25]. Interestingly, there were also some cases in which the indication for one or both interacting drugs was questioned and needed verification, an issue often encountered for PPIs [26]. These results may illustrate the importance of continuous medical reconsideration of drug treatment in relation to a patient’s evolving health status.

Alerts of *B* interactions were as common as *C* interactions. The most frequent *B* interaction alert concerned calcium and omeprazole, a combination that may impair the absorption of calcium and thereby contribute to an increased risk of fractures [27]. None of the *B* interactions was considered sufficient to justify any further medical action. This may not be surprising as *B* interactions are defined as having a clinical significance that either is “unknown” or “varies”.

According to Swedish regulations, each unit bag within a multi-dose drug dispensing system must be screened for drug interactions by a supervisory pharmacist [28], using the same drug interaction database (*J*anusmed) that automatically provides alerts for the prescribing physician. Despite this extra monitoring, the present study shows that one in eight interaction alerts for patients using this system could trigger a medical action. The fact that patients with multi-dose drug dispensing use many drugs may contribute to these findings. This system has indeed been associated with a rising number of drugs in the medication list [29], thereby increasing the potential for drug interactions [30]. Be that as it may, our results suggest that, despite control by the pharmacist, physicians need to pay attention to interaction alerts also among patients with multi-dose drug dispensing.

Our finding that only one in eleven interaction alerts seems to warrant further medical action for a specific patient suggests a need for caution in the interpretation of studies where quality of prescribing is equated with the surrogate measure “interaction alerts in a drug interaction database”. Including such alerts as measures in core outcome sets for the evaluation of interventions to improve prescribing practices, as recently proposed [13], could therefore be questioned.

## Strengths and limitations

To the best of our knowledge, this is the first study to provide information on additional medically justified actions in response to drug interaction alerts from a computerised decision support system among older patients in primary care. An important strength of the study is the comprehensive drug treatment assessments performed, first independently and then in consensus, by two physicians specialised in clinical pharmacology and/or family medicine. This approach ensures that the results are relevant from a medical perspective. Nevertheless, the weak inter-rater agreement illustrates the complexity of pharmacotherapeutic assessments, as recently discussed [31]. Another strength is that warranted alert-related actions are described in detail.

As we included consecutive patients, from one urban and one rural primary health care centre staffed by more than 20 physicians at different career stages [15], the results are likely to be acceptably generalisable for older patents in primary care. However, differing prescribing practices within and between countries may have implications for the external validity. Nevertheless, frequent interaction alerts involving, for instance, metal ions potentially interacting with absorption and drug combinations resulting in an increased risk of bleeding, are in agreement with those previously reported [22]. Another aspect worth mentioning is that there are several available information resources for drug interactions, and these have been reported not to be entirely consistent [32–34].

It may be regarded as a limitation that the assessors were restricted to the information available in the primary care medical records; although this included hospital discharge summaries [15], all relevant information may not have been available. Therefore, there may be undocumented reasons for the prescribing physician not to act on the unaddressed alerts. Another limitation is that *J*anusmed, in accordance with other established decision support sources for potentially problematic drug interactions like Lexicomp, Micromedex, and Stockley’s Drug Interactions, provides information only for pairs of drugs, i.e. not for all drugs combined [35–37]. Indeed, a significant proportion of drug interaction queries to a drug information centre, where the entire medication list for a specific patient is usually considered, yielded advice for clinical action although no *J*anusmed alert was triggered [38]. Interestingly, a Swedish decision support system to guide clinicians regarding combined effects of multiple medicines is currently under development [39]. An additional limitation may be that *J*anusmed primarily covers pharmacokinetic interactions and therefore pharmacodynamic interactions may be underrepresented among the alerts. In the present study, however, an underlying pharmacodynamic mechanism was described in 81 (44%) alerted drug-drug pairs (data not shown). Finally, it must be stressed that this study does not evaluate the value of decision support regarding potential drug interactions at the initiation of drug treatment. Indeed, as we did not evaluate the drug treatment longitudinally, no detailed information regarding the prescribing physician’s management of interaction alerts could be provided.

## Conclusion

This study shows that, using a computerised decision support system for drug interactions, alerts can be expected to be presented in more than every other older patient with two or more drugs in their medication list. Most of the alerts were already being addressed in health care, for instance by the monitoring of clinical or laboratory parameters, or were not relevant in the clinical setting. In about one in ten alerts, however, it may be appropriate to take further action, although, for unknown reasons, such steps were not taken. The underlying reasons for these alerts remaining unaddressed could be worth further investigation. As a minority of the alerts warrant medical action, interaction alerts seem to be of questionable value as indicators of problematic prescribing.

## Data Availability

The datasets generated and analysed during the current study are not publicly available owing to Swedish data protection laws. The data can be shared with authorised persons after approved application from the Swedish Ethical Review Authority (https://etikprovningsmyndigheten.se).

## Appendix 1

Guiding sentences for the assessment of whether an action related to the drug treatment, including the drug interactions alerted for, was medically justified at the individual level.

**Table:**

1	I would not change anything in the patient’s drug treatment
2	I would reconsider the drug treatment in the long term but would do nothing during the current visit; it could be reassessed at the next regular consultation
3	I would take some action to be able to make a decision regarding the drug treatment, for instance order a laboratory test, get more information about the patient or schedule an extra visit, before the next regular consultation
4	I would change the drug treatment at the index visit

Sentences (3) and (4) were collapsed into one category where some actions were considered medically justified prior to the next regular visit, whereas sentences (1) and (2) reflected that no action was required.

Taken from: Parodi López N, Svensson SA, Wallerstedt SM. Association between recorded medication reviews in primary care and adequate drug treatment management – a cross-sectional study. Scand J Prim Health Care. 2021; 39:419-428. 10.1080/02813432.2021.1973239.
